# Role of antimicrobial photodynamic therapy (aPDT) and systemic resveratrol on immediate implant placement in type 2 diabetic rats

**DOI:** 10.1111/php.70046

**Published:** 2025-10-24

**Authors:** Letícia Pitol‐Palin, Carolina Sayuri Wajima, Fábio Roberto de Souza Batista, Naara Gabriela Monteiro, Isadora Castaldi Sousa, Valdir Gouveia Garcia, Dóris Hissako Matsushita, Letícia Helena Theodoro, Roberta Okamoto

**Affiliations:** ^1^ Department of Diagnostic and Surgery and Dental Assistance Center for People with Disabilities (CAOE) Araçatuba Dental School, São Paulo State University (UNESP) São Paulo Brazil; ^2^ Department of Preventive and Restorative Dentistry Araçatuba Dental School, São Paulo State University (UNESP) São Paulo Brazil; ^3^ Department of Diagnostic and Surgery Araçatuba Dental School, São Paulo State University (UNESP) São Paulo Brazil; ^4^ Department of Basic Science Araçatuba Dental School, São Paulo State University (UNESP) São Paulo Brazil; ^5^ Latin American Institute of Dental Research and Education (ILAPEO) Curitiba Brazil; ^6^ Department of Basic Science and Dental Assistance Center for People with Disabilities (CAOE) Araçatuba Dental School, São Paulo State University (UNESP) São Paulo Brazil

**Keywords:** animal model, dental implants, diabetes mellitus, type 2, photochemotherapy, resveratrol

## Abstract

This study investigated the synergistic effects of resveratrol and antimicrobial photodynamic therapy (aPDT) on peri‐implant bone repair in a type 2 diabetes model. Forty‐eight rats were allocated into four groups: normoglycemic, normoglycemic + resveratrol, type 2 diabetes (T2D), and T2D + resveratrol. Diabetes was induced using a cafeteria diet and streptozotocin (35 mg/kg). Resveratrol (100 mg/kg) was administered systemically beginning 7 days later. After 14 days, maxillary molars were extracted, and surgical drilling was performed. Half of the animals in each group received aPDT (methylene blue and 660 nm diode laser) before immediate implant placement. Animals were euthanized 28 days post‐surgery for biomechanical, RT‐PCR, and confocal microscopy analyses. Resveratrol improved glycemic control and body weight. In T2D animals, aPDT significantly enhanced implant removal torque. Gene expression analyses revealed downregulation of bone resorption markers and upregulation of bone mineralization genes in T2D and T2D + resveratrol groups treated with aPDT. Confocal microscopy demonstrated increased mineral apposition rates in animals treated with resveratrol and/or aPDT. These findings suggest that the combination of systemic resveratrol and local aPDT enhances peri‐implant bone healing under diabetic conditions, highlighting a potential therapeutic approach to improve implant osseointegration in compromised metabolic states.

## Graphical abstract

This study evaluated the combined effects of antimicrobial photodynamic therapy (aPDT) and systemic resveratrol on bone repair around immediately placed implants in type 2 diabetic rats. Resveratrol improved glycemic control and body weight, while aPDT increased implant stability and bone mineralization. Gene expression and confocal analyses showed reduced bone resorption and enhanced mineral apposition when both treatments were applied. These results suggest that the association of resveratrol and aPDT promotes peri‐implant bone healing in diabetic conditions, offering a promising therapeutic strategy to enhance osseointegration in compromised metabolism.

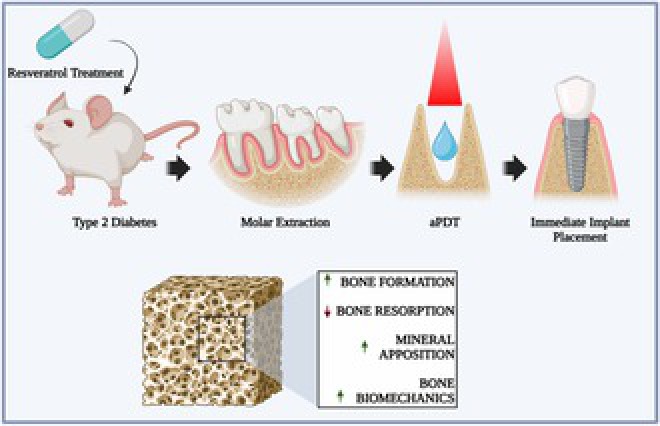

**Abbreviations**
aPDT: antimicrobial photodynamic therapy
cm: centimeters
IBSP: Integrin‐binding sialoprotein
mm: millimeters
N/cm: Newtons/centimeter
NG: normoglycemic group
NGrvt: Normoglycemic group treated with resveratrol
RT‐qPCR: Real time polymerase chain reaction
STZ: streptozotocin
T2D: Type 2 diabetes group
T2Drvt: Type 2 diabetes group treated with resveratrol
TRAP: Tartrate‐resistant acid phosphatase
vs.: versus
β‐Actin: Beta actin

## INTRODUCTION

Type 2 diabetes affects from 90% to 95% of diabetic patients^1^ and presents as a predisposing factor, an unhealthy lifestyle with foods rich in sugar, fat, and sodium, in combination with a lack of physical activity.^2^, ^3^, ^4^ Insulin resistance induced by obesity is usually linked to other disorders such as inflammation, hepatic steatosis, and oxidative stress.^3^ In addition to the damage to the systemic condition, dental complications are observed such as periodontal disease, bone loss, loss of teeth, and other types of infections that trigger a series of disorders for the dentist,^5^ such as the loss of implants related to the greater chance of the occurrence of infections that delay tissue repair,^5^, ^6^ cellular proliferation,^7^, ^8^ collagen metabolism,^6^, ^8^, ^9^ impairing the alveolar bone repair.^6^

Hyperglycemia and the presence of T2D are factors that must be taken into consideration during rehabilitation with dental implants^10^, ^11^ due to the high failure rate in the treatment of patients with this systemic condition. For successful osseointegration, some factors must be taken into account: the migration of inflammatory cells, cell proliferation with the creation of a new extracellular matrix, and finally, the formation and remodeling of this newly formed matrix.^11^ Phytotherapeutic compounds such as resveratrol (trans‐3,5,4′‐trihydroxystilbene) have been a widely accepted choice for the treatment of type 2 diabetes because it prevents glucose intolerance, has a powerful anti‐inflammatory effect, and can improve insulin sensitivity.^12^, ^13^, ^14^, ^15^ In addition, it facilitates the glucose transport from the blood into the cells and acts in the gastrointestinal tract, attenuating the glucose peak related to food ingestion.^12^, ^13^, ^14^, ^15^ This polyphenol is typically found in purple grape peel, in wines, and in some other plants.^12^ Like some hypoglycemics,^16^, ^17^ resveratrol is also beneficial to bone tissue by preventing bone loss, helping in post‐surgical bone repair,^16^, ^17^ and promoting osteoblastogenesis besides potentiating the action of vitamin D receptors.^12^, ^13^, ^14^

The use of antimicrobial photodynamic therapy (aPDT), which consists of the association of a photosensitizing agent with a light source, such as a low‐level laser, is consolidated as promising for the treatment of dental complications by exerting positive effects at the molecular, cellular, and tissue levels.^18^, ^19^, ^20^, ^21^ Methylene blue, as a photosensitizer, exerts broad‐spectrum antimicrobial effects besides having easy tissue absorption.^18^, ^19^, ^20^, ^21^, ^22^, ^23^, ^24^ aPDT favors bone repair, being an effective treatment to control infections,^18^, ^20^ accelerating the process of tissue repair and minimizing undesirable symptoms and postoperative complications.^18^, ^19^, ^20^, ^21^, ^22^, ^23^, ^24^ The versatility in the use of aPDT allows the exploration of several protocols, varying the number of sessions, irradiation parameters, and types of photosensitizers according to the results that are aimed to achieve.

Considering that no previous studies have evaluated the combination of these therapies, the main hypothesis of this study is that the association between two non‐invasive treatments such as resveratrol and the local use of aPDT would work to prevent implant loss in T2D. Due to their action mechanisms acting in different pathways of the organism (local or systemic), which would promote support throughout the process of peri‐implant bone repair. Thus, the aim of this study was to evaluate the synergistic effect of resveratrol treatment and antimicrobial photodynamic therapy (aPDT) on peri‐implant bone repair in a type 2 diabetes model.

## MATERIALS AND METHODS

### Animals

The study was approved by the Research Ethics Committee of Araçatuba Dental School, under number 00153‐2019, following the Animal Research N3CR guidelines for Reporting of In Vivo Experiments (ARRIVE) guidelines.^25^ Forty‐eight male rats (*Rattus norvegicus* albinus, Wistar), weighing 300 g and 3 months old, were divided into four groups: normoglycemic (NG; *n* = 12); normoglycemic + resveratrol (NGrvt; *n* = 12); type 2 diabetes (T2D; *n* = 12); and type 2 diabetes + resveratrol (T2Drvt; *n* = 12), where half of the specimens in each group received aPDT (*n* = 6) and the other half had no local therapy (*n* = 6). The animals were kept in cages in a stable temperature environment (22°C ± 2°C, light control cycle 12 light hours, 12 h dark) and a balanced diet (NUVILAB, 1.4% Ca and 0.8% P + water ad libitum).

The sample size for each group was determined using the power test through the website http://www.openepi.com/SampleSize/SSMean.htm (OpenEpi, Version 3, open‐source calculator), based on previous results already published^6^, ^26^: the averages obtained by the results used for the calculation were 3.06 and 4.898 and the standard deviations were 0.26 and 0.024, with a significance level of 5% and a power of 95% in a one‐tailed hypothesis test. The animals were identified by numbers and randomly separated by Microsoft Office Excel software (Microsoft, Redmond, WA, USA), respecting a 1:1 allocation rate for each group. After randomization of the animals, one maxilla was used to perform removal torque and RT‐PCR, while the opposite side was selected to perform confocal microscopy analysis.

### Diabetes induction

The animals were fed with a cafeteria diet, water with sugar, and water ad libitum. Cafeteria diet causes insulin resistance, obesity, metabolic syndrome, and mimics the poor diet consumed by the world population.^3^, ^4^, ^6^ The cafeteria diet (Table 1) was made with stuffed crackers, wafer crackers, corn chips, and a bottle of water with a concentration of 12% sucrose. Each animal was weighed daily with 30 g of food.^6^ After 3 weeks of cafeteria diet, the T2D animals were anesthetized by intramuscular infiltration of 5 mg/kg xylazine hydrochloride (Dopaser® – Laboratórios Calier do Brazil Ltda., Osasco, SP, Brazil) and 50 mg/kg ketamine hydrochloride (Vetaset® – Fort Dodge Animal Health Ltda., Campinas, SP, Brazil). Antisepsis of the scrotum region was performed with 70% alcohol, and in the T2D and T2DR rats, a low dose of streptozotocin (STZ – Sigma©, Merck KGaA, Darmstadt, Germany and/or its affiliates) was injected by penile vein (35 mg/kg) dissolved in vehicle (sodium citrate solution 0.1 M, pH = 4.5).^6^ Diabetes confirmation was made 1 week after T2D induction, following the criteria: glucose concentration greater than 198 mg/dL after 120 min of oral glucose solution.^6^ Body weight and blood glucose were measured once a week until euthanasia.

**TABLE 1:** Cafeteria diet composition for T2D and T2Drvt groups.

Food item	Manufacturer	Amount	Kcal	Tot. fat (%)	Sat. fat (%)	Trans fat (%)	Carb. (%)	Prot. (%)	Sod. (mg)	Chol. (g)
Stuffed crackers	Zabet	10 g	139.0	12.0	12.0	0.0	7.0	2.0	66.0	0.0
Wafer crackers	Bauducco	10 g	138.0	14.0	14.0	0.0	6.0	2.0	64.0	0.0
Corn chips	Keleck	10 g	92.0	2.0	2.0	0.0	6.0	2.0	229.0	0.0
Water with sugar (12%)	União	200 mL	120.0	0.0	0.0	0.0	2.0	0.0	0.0	0.0

*Note*: Each animal received 30 g of each type of food daily. The kilocalories (Kcal), total fat (Tot. Fat), saturated fat (Sat. Fat), trans fat (Trans Fat), carbohydrate (Carb.), protein (Prot.), sodium (Sod.), and cholesterol (Chol.) values were provided by a nutritional table according to the manufacturers.

### Resveratrol treatment

After T2D confirmation, the daily treatment with resveratrol was started for NGrvt and T2Drvt animals until euthanasia. For this, the animals received, by oral gavage, resveratrol (100 mg/kg/day, Aphoticário Pharmacy of Manipulation Ltda, Araçatuba, São Paulo, Brazil) dissolved in dimethyl sulfoxide (DMSO).^27^ DMSO is chosen for its cryopreservation properties, which make it essential for use in pharmacies and therapies. It also has the ability to combine with various types of acids, carbohydrates, lipids, and various other substances without permanently changing their molecular composition and has no cytotoxic effects on tissues. NG and T2D animals received only the vehicle (DMSO).

### Immediate implant placement

Fourteen days after the beginning of resveratrol treatment, all animals were fasted for 8 h before the surgical procedure and sedated by the combination of ketamine and xylazine hydrochloride. After the sedation, the animals were submitted to dental extraction of the first upper molars and, immediately, the drilling was performed with a 1.1 mm diameter helical drill coupled with a counter‐angle with a 20:1 reduction (Angular Part 3624N 1:4, Head 67RIC 1:4, KaVo®, Kaltenbach & Voigt GmbH & Co, Biberach, Germany), mounted on an electric motor (BLM 600®; Driller, São Paulo, SP, Brazil), at a speed of 1000 rpm, under irrigation with isotonic 0.9% sodium chloride solution (Fisiológico®, Laboratórios Biosintética Ltda®, Ribeirão Preto, SP, Brazil) and a depth of 3.0 millimeters.^28^

After the creation of the surgical sites, half of the animals of each group received as immediate treatment the aPDT, and the other half did not receive any treatment (NT). For the animals that received aPDT, a quantity of 0.03 mL of methylene blue (100 μg/mL, Aphoticário Pharmacy of Manipulation Ltda, Araçatuba, São Paulo, Brazil) was deposited for 60 s. The volume of 0.03 mL corresponded to the capacity of the surgical site and was sufficient to fill the implant bed without overflow. The concentration of 100 μg/mL was selected based on previous reports demonstrating effective antimicrobial action in vivo while maintaining biocompatibility.^20^, ^21^ Then, the surgical site was irradiated with Indium Gallium Aluminum Phosphorous (InGaAlP) diode laser (660 nm; Thera Lase®, DMC Equipment Ltda®, SP, Brazil) with 0.0283 cm^2^ of spot area, with the following parameters: power of 35 mW; continuous operation mode; energy of 2.1 J; time of irradiation of 60 s; energy density of 74.2 J/cm^2^; irradiance of 1.23 W/cm^2^.^21^ The laser optical fiber was positioned at a single point in the center of the implant installation site, parallel to its long axis and in contact with the surgical local prior to the implant installation. The surgical site of the NT animals did not receive any treatment, only irrigation with 0.9% sodium chloride solution before the implant installation.

**FIGURE 1:**
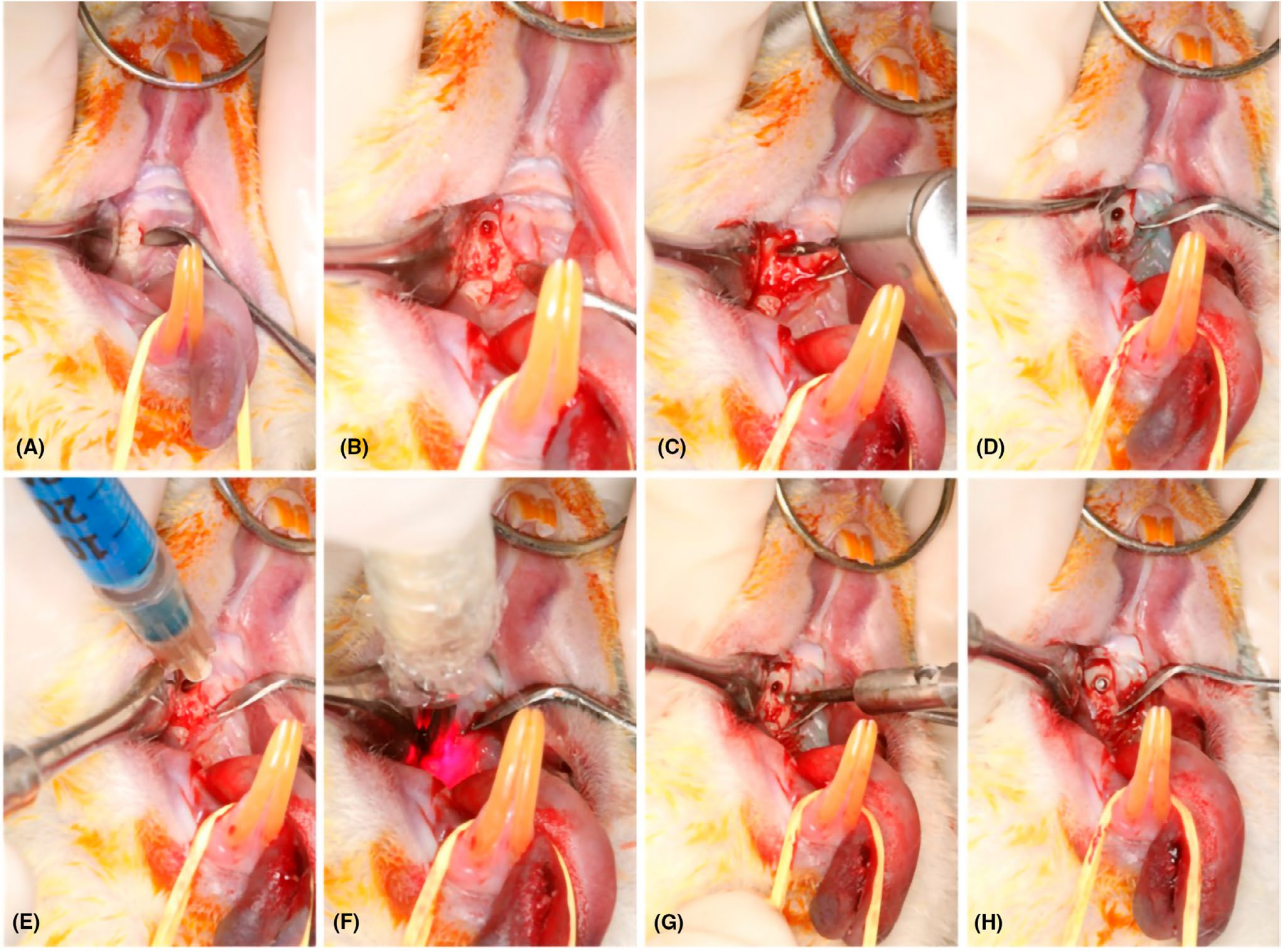
Representative images for the clinical aspect of implant installation with aPDT: (A) Beginning of syndesmotomy with the aid of a Hollenbeck 3S sculptor; (B) dental extraction; (C) drilling and preparation of the surgical site; (D) surgical site aspect; (E) application of methylene blue for 60 s; (F) low‐power laser irradiation for 60 s; (G) surgical local prepared for implant installation; (H) final aspect of implant installation.

Immediately after treatments, each animal received one implant at the right and left first upper molar region.^28^ The titanium implants were custom produced by Medens (Medens Implants, Itu—SP, Brazil), with commercially pure titanium grade IV, based on the concept of double acid etching, with a diameter of 1.4 mm and a length of 2.7 mm, sterilized by gamma rays, using the same processes as those commercially available. The tissue closure was done with monofilament wire (Nylon 5.0, Ethicon, Johnson, São José dos Campos, Brazil). In the immediate postoperative period, each animal received a single intramuscular dose of penicillin G‐benzathine (Pentabiotic Small Veterinary, Fort Dodge Saúde Animal Ltda., Campinas, São Paulo, Brazil) and analgesia with Sodium Dipyrone (1 mg/kg/day, Ariston Indústrias Químicas e Farmacêuticas Ltda, São Paulo, Brazil) (Figure 1).

**FIGURE 2:**
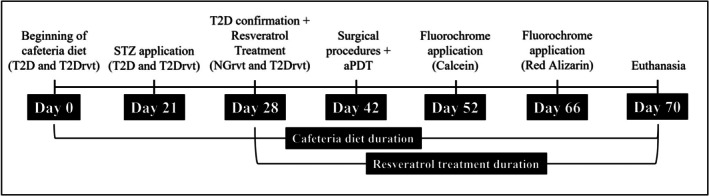
Experimental model timeline.

After 10 days, all animals received fluorochrome calcein intramuscularly (20 mg/kg). Alizarin red fluorochrome was also used intramuscularly at a dose of 20 mg/kg for each animal 14 days after calcein, according to previous studies.^6^ The animals were euthanized 28 days after the implant surgery (Figure 2).

### Biomechanical analysis (removal torque)

The animals were euthanized by anesthetic overdosage of xylazine hydrochloride (Dopaser®—Laboratórios Calier do Brazil Ltda., Osasco, Brazil) and ketamine hydrochloride (Vetaset®—Fort Dodge Animal Health Ltd.a., Campinas, Brazil) 28 days after the implant installation. The jaws were accessed to expose the implants, and a digital torquemeter (Homis, São Paulo, SP, Brazil) coupled with a 0.9 mm hexagonal digital key (Medens, São Paulo, SP, Brazil) was used. An anti‐clockwise movement was applied until the rotation of the implant inside the bone tissue and the complete rupture of the bone/implant interface. The maximum value for the rupture was recorded on the torquemeter (N.cm) and noted.^28^, ^29^

### Real time polymerase chain reaction (RT‐qPCR)

After the removal torque analysis, the bone attached to the implants was collected for molecular biology experiments, and RT‐PCR was performed to evaluate the gene expression of markers related to the peri‐implant bone remodeling process. Each bone fragment was washed in PBS and subsequently frozen in liquid nitrogen for total RNA extraction with Trizol reagent (Life Technologies Invitrogen, Carlsbad, CA, USA) and Kit Promega (Promega® Corporation, Madison, Wisconsin, USA). RNA was quantified on a Molecular Devices Spectramax 190® for analysis of RNA integrity, purity, and concentration. The cDNA was prepared using 1 μg of RNA in a reverse transcriptase reaction (M‐MLV reverse transcriptase; Promega® Corporation, Madison, WI, USA). The cDNA was aliquoted and added to 10 μL of Taqman® Fast Advanced Mastermix (Applied Biosystems®, Foster City, CA, USA), 1 μL of Taqman® Gene Expression Assays (Applied Biosystems®, Foster City, CA, USA), and RNAse‐free water to obtain 20 μL of solution. The amplifications were performed in duplicate in 96‐well plates.

Primers and probes (Table 2) from the Taqman® Gene Expression Assays system were obtained commercially to evaluate gene expression of Ibsp (integrin‐binding sialoprotein/IBSP—Rn01450118_m1) and Trap1 (tartrate‐resistant acid phosphatase/TRAP—Rn01424636_m1). StepOne Plus® (Applied Biosystems®, Foster City, CA, USA) was used to perform the amplification and detection reactions under the conditions: 50°C (2 min), 95°C (10 min), and 40 cycles of 95°C (15 s), 60°C (1 min), followed by a standard denaturation curve. β‐Actin and b2m were used as housekeeping genes; the relative gene expression was calculated by the 2−ΔΔCT method, and β‐actin was selected for normalization based on its cycle threshold value.^30^, ^31^

**TABLE 2:** TaqMan probes for RT‐PCR.

Gene	Gene name	Identification
Ibsp	Integrin‐binding sialoprotein	Rn01450118_m1
Trap	Tartrate‐resistant acid phosphatase	Rn01424636_m1
β‐Actin	Beta Actin	Rn0067869_m1

### Confocal microscopy analysis

The opposing maxillae for the removal torque analysis were utilized for the confocal microscopy analysis. The samples were dehydrated in increasing concentrations of alcohols, then imbibed and infiltrated in a 1:1 solution of acetone and slow methyl methacrylate (PMMAL) (Classical, Classical Dental Articles, São Paulo, Brazil). Subsequently, they were given 3 PMMAL baths, and in the last bath, the 1% benzoyl peroxide catalyst (Riedel—De Haën AG, Seelze—Hannover, Germany) was added. The latter bath (PMMAL and catalyst) was performed with the pieces placed in capped glass vials, which were held at a temperature of 37°C for 5 days until the resin was polymerized. After polymerization, the blocks containing the specimens were initially reduced with a “Maxcut” drill mounted on a Kota bench motor (Strong 210, São Paulo, Brazil), parallel to the long axis of the hemimaxilla (sagittal plane). Progressive manual wear on an automatic polisher (ECOMET 250PRO/AUTOMET 250, Buehler, Lake Bluff, IL, USA) was used. The sections were mounted on histological slides. After that, the sections were analyzed at a Leica CTR 4000 CS SPE microscope (Leica Microsystems, Heidelberg, Germany) under a 10x objective (original magnification 100). Images of calcein and alizarin red fluorochromes representing old bone and new bone, respectively, were obtained separately (bone dynamics).^6^ Finally, these images were reconstructed, providing the overlap of the fluorochromes that were used, to evaluate bone turnover by the mineral apposition rate (MAR).^26^ The images were transported to the image analyzer software, ImageJ (Image Processing and Analysis Software, Bethesda, MD, USA). Through the free hand tool, the area of fluorochrome precipitation (calcein/alizarin) was measured. Five measurements were drawn with the straight tool that extended from the outer margin of the calcein toward the outer margin of the alizarin. The obtained value was divided by 10, which represented the interval of days between the injections of both fluorochromes.^32^

### Statistical analysis

For statistical analysis, GraphPad Prism 7.03 (GraphPad Software, La Jolla, USA) was used. The homoscedasticity was assessed via the Shapiro–Wilk test to distinguish the parametric and non‐parametric data according to the distribution of quantitative results in the normality curve, and the ANOVA two‐way test was chosen in order to determine if there were differences among the groups, considering factors related to systemic treatment or local treatment. Holm–Sidak post‐test was performed in order to indicate the differences in the direct comparisons of the groups. A significance level of 5% was considered for all tests. The following differences were evaluated: Intergroup—Groups (NG, NGrvt, T2D, and T2Drvt) vs. NT; Groups vs. aPDT; and Intragroup—NT vs. aPDT.

## RESULTS

### Clinical data (glycemic level and body weight)

Comparing the groups, it was observed that the cafeteria diet elevates circulating glucose levels higher than 100 mg/dL in the initial weeks of the experiment, resulting in insulin resistance in T2D and T2Drvt animals. The association of STZ application leads to progression in the type 2 diabetes condition, resulting in increased blood glucose values that enable the diagnosis of diabetes in the animals. Moreover, the use of systemic resveratrol proved effective in supporting the treatment of type 2 diabetes, reducing glucose levels and achieving glycemic stability in normoglycemic rats. At the end of the experiment, daily resveratrol use resulted in decreased glycemia in T2Drvt animals when compared to T2D animals (Table 3). Over the course of the experiment, the NG animals gained an average of 100 g, while the NGrvt animals gained an average of 60 g. This fact suggests that the continuous use of resveratrol is an ally in the maintenance of body weight, avoiding weight gain. T2D animals had an initial weight gain due to the cafeteria diet, but after the diabetes induction, there was an average loss of 80 g due to their systemic debilitation until the end of the experiment. In contrast, the systemic use of resveratrol for T2Drvt maintained the weight of the animals even with the presence of systemic interference (Table 4).

**TABLE 3:** Clinical data of glycemia (mean and standard deviation) in NG, NGrvt, T2D, and T2Drvt groups in each period.

	Initial	STZ application	Resveratrol treatment	Surgical procedures	Euthanasia
NG	75.7 ± 6.58^a^	92.7 ± 7.30^a^	88.5 ± 6.34^a^	77.7 ± 6.05^a^	97.3 ± 6.86^a^
NGrvt	85.5 ± 7.79^b^	79.75 ± 5.11^a^	102.8 ± 9.86^a^	95.75 ± 11.12^a^	90.25 ± 4.41^a^
T2D	98.3 ± 6.25^c^	125.5 ± 14.12^b^	384.8 ± 83.77^b^	366.6 ± 61.84^b^	412.2 ± 47.54^b^
T2Drvt	95.25 ± 5.06^c^	127.9 ± 29.72^b^	370.9 ± 61.11^b^	318.8 ± 42.23^c^	227.9 ± 39.93^c^

*Note*: Glycemic values higher than 198 mg/dL indicate the T2D development. The statistically significant differences in each analyzed period are represented by different lowercase letters (a, b, c, d). Two‐way ANOVA statistical test (*p* < 0.05).

**TABLE 4:** Clinical data of body weight (mean and standard deviation) in NG, NGrvt, T2D, and T2Drvt groups throughout the experimental design.

	Initial	STZ application	Resveratrol treatment	Surgical procedures	Euthanasia
NG	374.5 ± 22.53^a^	401.5 ± 22.86^a^	415.4 ± 21.1^a^	432.3 ± 21.56^a^	478.9 ± 23.73^a^
NGrvt	341.7 ± 25.74^b^	360.5 ± 21.93^b^	366.7 ± 23.16^b^	376.5 ± 27.92^b^	401.2 ± 24.73^b^
T2D	427.7 ± 12.33^c^	501.0 ± 32.15^c^	476.3 ± 40.16^c^	447.3 ± 42.21^a^	419.5 ± 62.61^ab^
T2Drvt	435.5 ± 36.91^d^	493.1 ± 55.84^c^	483.9 ± 57.21^c^	481.4 ± 53.87^a^	517.0 ± 80.43^ac^

*Note*: The statistically significant differences in each analyzed period are represented by different lowercase letters (a, b, c, d). ANOVA two‐way statistical test (*p* < 0.05).

### Biomechanical analysis (removal torque)

**FIGURE 3:**
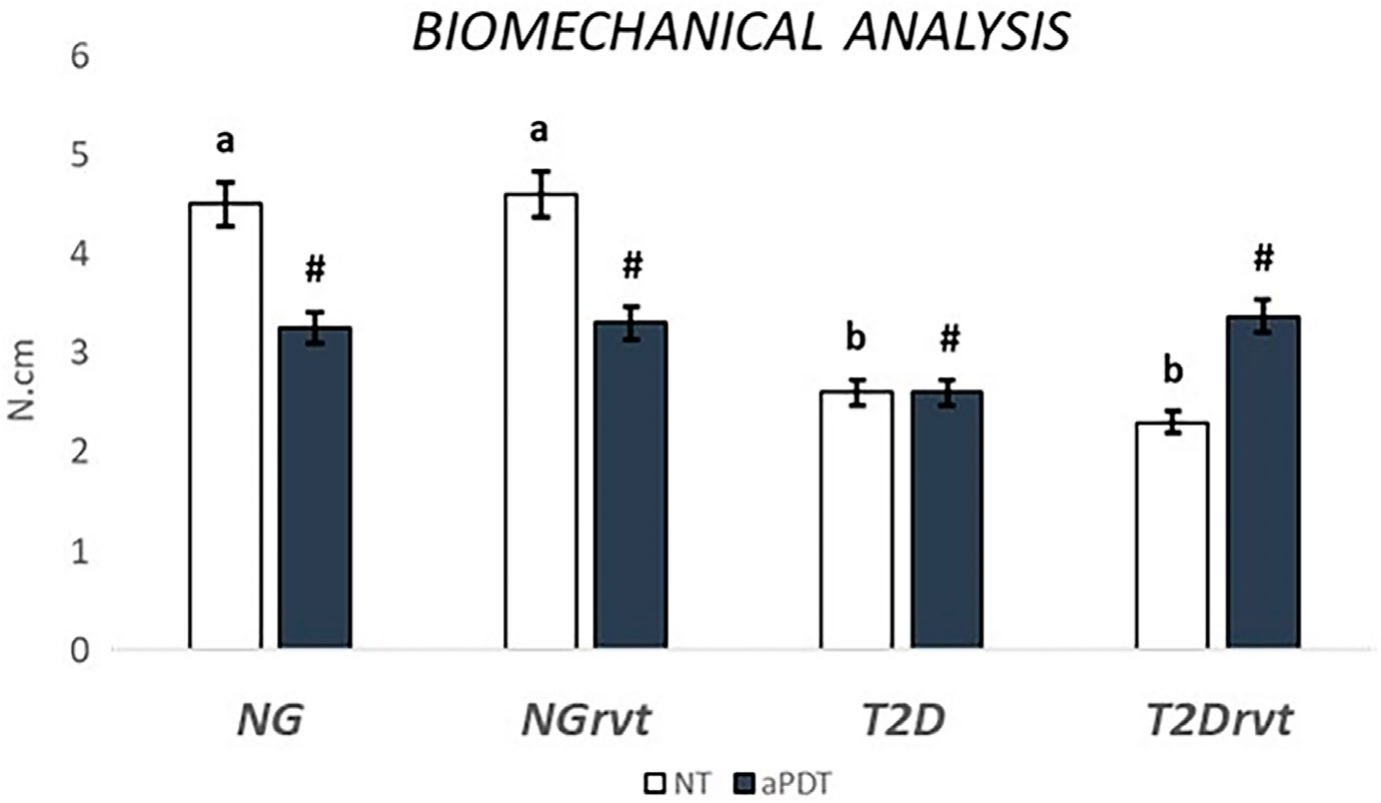
Graph of removal torque results. ANOVA two‐way statistical test: Groups vs. NT (significant differences represented by different lowercase letters in the graphs); Groups vs. aPDT (significant differences represented by different symbols [#] in the graphs); and Intragroup—NT vs. aPDT showed no statistically significant difference (*p* > 0.05).

The comparison between groups vs. *NT* showed a statistically significant difference when comparing the NG and NGrvt groups vs. T2D and T2Drvt, where NG‐NT and NGrvt‐NT had higher removal torque values than T2D‐NT and T2Drvt‐NT (*Groups* vs. *NT*—NG vs. T2D: *p* = 0.0065; NG vs. T2Drv: *p* = 0.0034; NGrvt vs. T2D: *p* = 0.0149; NGrvt vs. T2Drvt: *p* = 0.0076). The comparison between groups vs. *aPDT* showed no statistically significant difference (*p* > 0.05) between the systemic groups with the use of aPDT. Finally, the comparison intragroup showed no statistically significant difference (*p* > 0.05) between the removal torques in each group; however, the use of aPDT associated with systemic resveratrol in T2Drvt animals promoted an increase in implant removal torque (*p* < 0.05; Figure 3).

### Real‐time polymerase chain reaction (RT‐PCR)

#### Bone sialoprotein

**FIGURE 4:**
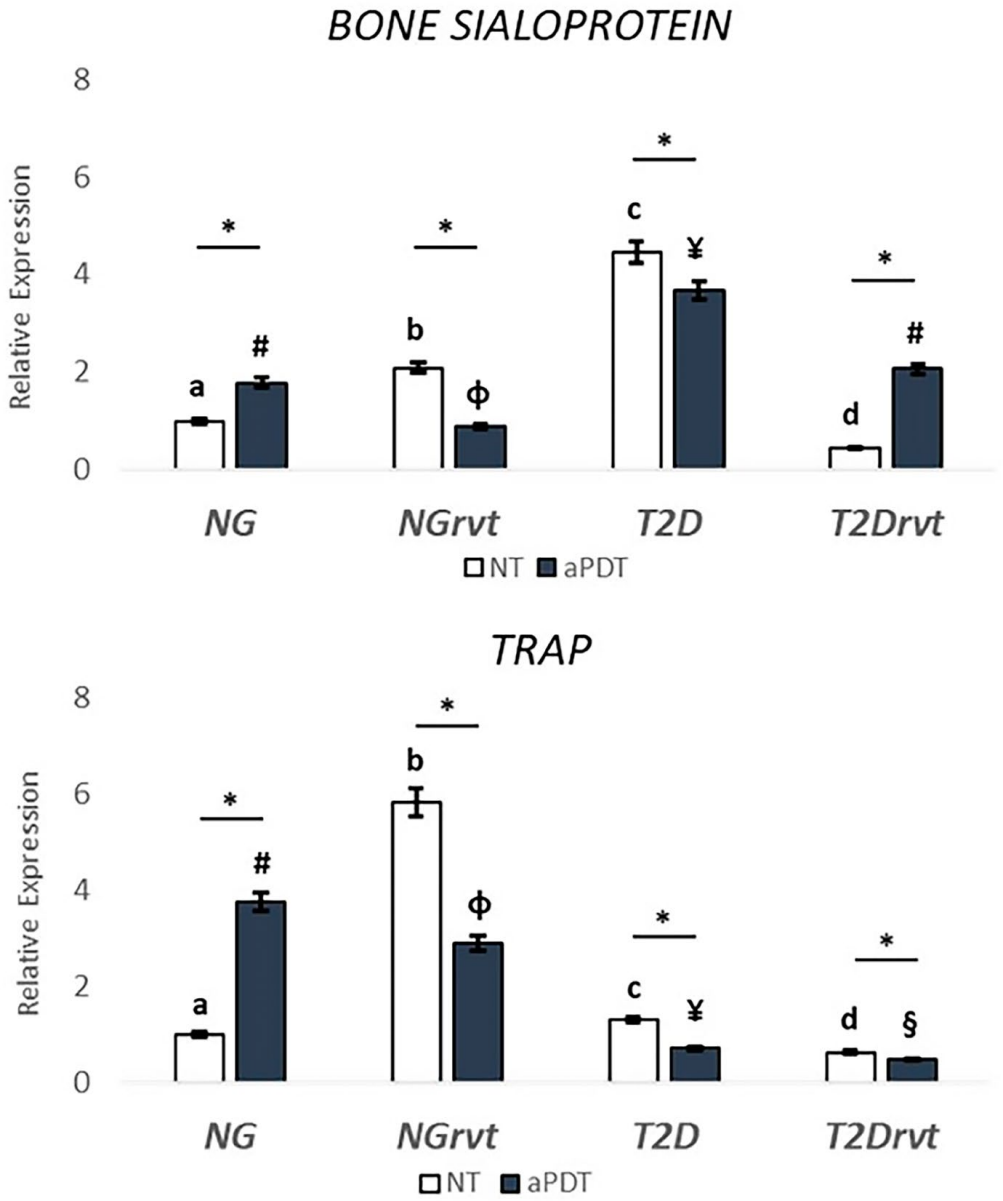
Graphs of the relative gene expressions of IBSP and TRAP. ANOVA two‐way statistical test (*p* < 0.05): Group vs. NT (significant differences represented by different lowercase letters in the graphs); Group vs. aPDT (significant differences represented by different symbols [#, ɸ, ¥, §] in the graphs); and Intragroup—NT vs. aPDT (significant differences represented by *).

The gene expression of IBSP in the comparison between groups vs. *NT* showed a statistically significant difference between all groups, where T2D‐NT showed higher numerical values (*Groups* vs. *NT* – NG vs. NGrvt: *p* < 0.0001; NG vs. T2D: *p* < 0.0001; NG vs. T2Drvt: *p* = 0.0036; NGrvt vs. T2D: *p* < 0.0001; NGrvt vs. T2Drvt: *p* < 0.0001; T2D vs. T2Drvt: *p* < 0.0001). The comparison between groups vs. *aPDT* showed a statistically significant difference when comparing NG‐aPDT and T2Drvt‐aPDT vs. NGrvt‐aPDT and T2D‐aPDT, and T2D‐aPDT presents higher gene expression (*Groups* vs. *aPDT* – NG vs. NGrv: *p* = 0.0003; NG vs. T2D: *p* < 0.0001; NGrvt vs. T2D: *p* < 0.0001; NGrvt vs. T2Drvt: *p* < 0.0001; T2D vs. T2Drvt: *p* < 0.0001). For intergroup comparison, gene expression of IBSP at 28 days showed statistically significant difference when comparing NT vs. aPDT in all systemic conditions (*NT* vs. *aPDT* – NG: *p* = 0.0006; NGrvt: *p* < 0.0001; T2D: *p* = 0.0006; T2Drvt: *p* < 0.0001) (Figure 4).

#### Tartrate‐resistant acid phosphatase

The TRAP relative gene expression showed a statistically significant difference in all comparisons between all groups at 28 days after implant installation in the maxilla (*Groups* vs. *NT* – NG vs. NGrvt: *p* < 0.0001; NG vs. T2D: *p* = 0.0003; NG vs. T2Drvt: *p* < 0.0001; NGrvt vs. T2D: *p* = 0.0107; NGrvt vs. T2Drvt: *p* < 0.0001; T2D vs. T2Drvt: *p* < 0.0001). The comparison between groups vs. aPDT showed a statistically significant difference between all the groups (*Groups* vs. *aPDT* – NG vs. NGrvt: *p* < 0.0001; NG vs. T2D: *p* < 0.0001; NG vs. T2Drvt: *p* < 0.0001; NGrvt vs. T2D: *p* < 0.0001; NGrvt vs. T2Drvt: *p* < 0.0001; T2D vs. T2Drvt: *p* = 0.0010). Furthermore, it is possible to observe that the use of aPDT at the time of implant installation promoted a decrease in TRAP gene expression when compared to the absence of local therapy (*NT* vs. *aPDT* – NG: *p* < 0.0001; NGrvt: *p* < 0.0001; T2D: *p* < 0.0001; T2Drvt: *p* = 0.0134) (Figure 4).

### Confocal microscopy analysis

#### Bone dynamics

**FIGURE 5:**
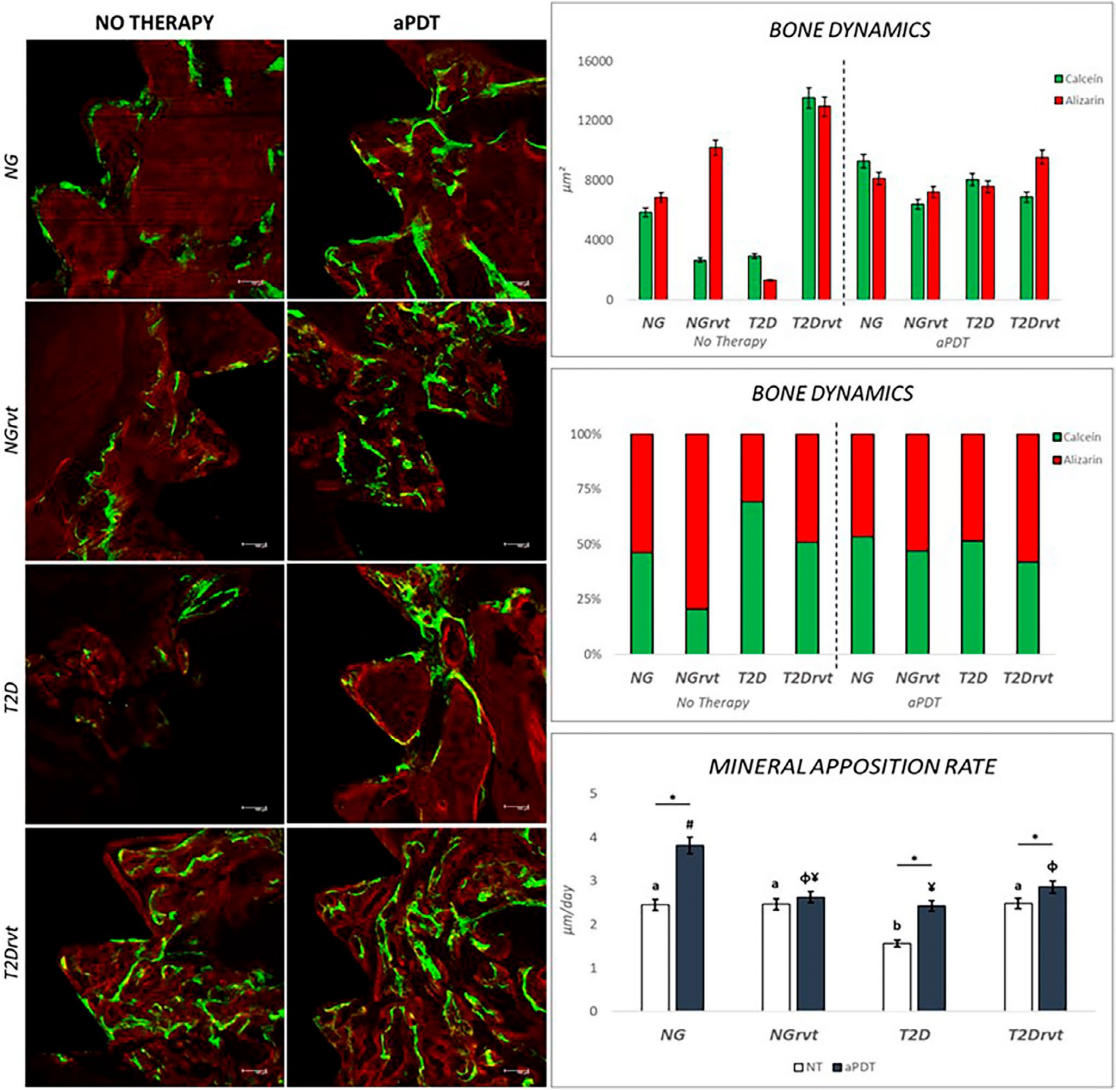
Confocal microscopy results of bone dynamics and mineral apposition rate. Representative photomicrographs of the NG, NGrvt, T2D, and T2Drvt groups in the comparison of NT vs. aPDT (10× magnification). *Bone Dynamics*: Green columns correspond to calcein values, while red columns correspond to alizarin red values. ANOVA two‐way statistical test (*p* < 0.05). MAR: Comparison of the daily mineral apposition rate in the groups. ANOVA two‐way statistical test (*p* < 0.05): Group vs. NT (represented by lowercase letters in the graphs); Group vs. aPDT (represented by symbols [#, ɸ, ¥] in the graphs); and Intragroup—NT vs. aPDT (represented by *).

The application of fluorochromes in two different periods makes it possible to analyze two different moments of the peri‐implant repair process. Thus, the results show that in the NG‐NT group there is a balance in the peri‐implant repair process, with the presence of mature bone (calcein) and neoformed bone (alizarin red). In NG‐aPDT, it is possible to observe the same standard, but when using local therapy there is an increase in the precipitation of fluorochromes. On the other hand, in NGrvt‐NT, it is possible to observe an increase in alizarin red precipitation and low calcein precipitation, indicating that in this group, there was a recent mineral matrix deposition. The use of aPDT in NGrvt‐aPDT promoted a balance in mineral matrix deposition, similar to the NG group (Figure 5; Table 5).

**TABLE 5:** Data of comparison of confocal microscopy analysis for the bone dynamics parameter between groups.

	Comparison	*p* value*
Calcein	NG‐NT vs. T2Drvt‐NT NG‐aPDT vs. NGrvt‐NT NG‐aPDT vs. T2D‐NT NG‐aPDT vs. T2Drvt‐NT NGrvt‐NT vs. T2D‐aPDT NGrvt‐NT vs. T2Drvt‐NT NGrvt‐NT vs. T2Drvt‐aPDT NGrvt‐aPDT vs. T2Drvt‐NT T2D‐NT vs. T2D‐aPDT T2D‐NT vs. T2Drvt‐NT T2Drvt‐NT vs. T2Drvt‐aPDT	0.0002 0.0009 0.0013 0.0479 0.0065 < 0.0001 0.0486 0.0004 0.0104 < 0.0001 0.0008
Alizarin red	NG‐NT vs. T2D‐NT NG‐NT vs. T2Drvt‐NT NG‐aPDT vs. T2D‐ST NG‐aPDT vs. T2Drvt‐NT NGrvt‐NT vs. T2D‐NT NGrvt‐aPDT vs. T2D‐NT NGrvt‐aPDT vs. T2Drvt‐NT T2D‐NT vs. T2D‐aPDT T2D‐NT vs. T2Drvt‐NT T2D‐NT vs. T2Drvt‐aPDT T2D‐aPDT vs. T2Drvt‐NT	0.0050 0.0020 0.0006 0.0174 <0.0001 0.0026 0.0038 0.0015 <0.0001 <0.0001 0.0068

*Note*: ANOVA two‐way statistical test.

* *p* < 0.05.

#### Mineralized apposition rate (MAR)

This analysis makes it possible to evaluate the daily rate of mineral deposition (μm/day) over the 28 days of peri‐implant repair. Thus, the comparison between *groups* vs. *NT* showed a statistically significant difference when comparing T2D‐NT to the other groups without local treatment, where the group with systemic interference presented a lower rate of mineral apposition over the days (*SI* vs. *NT* – T2D vs. NG: *p* = 0.0002; T2D vs. NGrvt: *p* = 0.0002; T2D vs. T2Drvt: *p* = 0.0001). For the comparison between *groups* vs. *aPDT*, it can be observed that the use of local therapy increased MAR in all systemic conditions, with a statistically significant difference between NG vs. NGrvt, T2D and T2Drvt and between T2D vs. T2Drvt (SI vs. aPDT – NG vs. NGrvt: *p* < 0.0001; NG vs. T2D: *p* < 0.0001; NG vs. T2Drvt: *p* = 0.0001; T2D vs. T2Drvt: *p* = 0.0075). Finally, when comparing the local therapies in each systemic condition, there was a statistically significant difference in the NG, T2D, and T2Drvt groups, where the use of aPDT promoted an increase in MAR (NT vs. aPDT – NG: *p* < 0.0001; T2D: *p* = 0.0006; T2Drvt: *p* = 0.0392) (Figure 5).

## DISCUSSION

A previous study^6^ showed that type 2 diabetes influences the increase in inflammatory response of alveolar bone repair, impairing collagen fiber maturation and delaying the mineralization process. This fact would have a significant impact on implant rehabilitation processes, in view of the impairment to the recipient site of these implants. Thus, this study evaluates the peri‐implant repair of type 2 diabetic animals using local and systemic therapies that contribute to reducing the inflammatory process, triggering an improvement in bone quality and biomechanics.

Resveratrol presents beneficial effects in terms of glucose and lipid homeostasis and reduction of body fat accumulation.^33^, ^34^ Systemically, its daily use was satisfactory for the reduction of glycemic levels in type 2 diabetic rats, but it does not replace the effects of hypoglycemic drugs as presented in other studies.^6^, ^33^ For NGrvt animals, the daily use of the phytotherapy treatment was effective for avoiding the increase in blood glucose levels throughout the experiment. After diabetes induction, a side effect present in animals is weight loss, and again, resveratrol was an ally for the systemic condition. Resveratrol prevented weight loss as well as restored the clinical conditions of T2Drvt rats, a fact directly related to the reduction of hyperglycemia in this group of animals.^33^, ^34^ The inverse occurred in NGrvt animals, where the continuous use of resveratrol caused a decrease in weight gain, promoting a maintenance of body and fat mass. Other studies^34^, ^35^, ^36^, ^37^ in vitro and in rodent models indicate that resveratrol can modulate the functions of adipogenesis, lipogenesis, lipolysis, thermogenesis, and fatty acid oxidation, a factor that may be related to the events in animals NGrvt and T2Drvt.

The continuous use of resveratrol in NGrvt‐NT and T2Drvt‐NT did not affect the removal torque of the animals compared to the animals without systemic treatment. It is worth noting that there are no studies in the literature, to date, where the administration of resveratrol has been evaluated in a maxillary implant installation model, considering that this is an environment in which there is exposure to bacteria, with no control of biofilm or periodontal condition. The performance of aPDT in groups NG and NGrvt did not promote effects on bone biomechanics, a fact that could be explained by the evaluation period of this study. On the other hand, for diabetic animals, aPDT or normal physiological conditions improved the bone biomechanical condition of implants at 28 days, especially in T2Drvt‐aPDT. These results suggest that the healthy body has bone repair conditions that do not depend on local support to assist it, as opposed to what occurred in animals with type 2 diabetes.

Local therapies that accelerate the peri‐implant repair process have received great emphasis in current dental studies, as in the case of topographical modifications^38^, ^39^, ^40^ to the implant surface by physical or chemical treatment to increase porosity, the addition of biocompatible coatings with pharmaceutical drugs, or the use of photonic therapies.^41^, ^42^ Resveratrol treatment in NGrvt‐NT caused an increase in IBSP and especially TRAP expression. The use of aPDT in the NG group promoted a decrease in mineralization‐related gene (IBSP) and an increase in genes related to osteoclastic activity and bone resorption (TRAP), when compared to animals that did not receive any local treatment. This fact may be related to the increased fluorochrome precipitation in this group, indicating that the molecular response was initiating the bone remodeling process. Resveratrol treatment in T2Drvt causes an increase in IBSP and a decrease in TRAP with the use of aPDT, which would indicate a decrease in osteoclastic activity and an increase in bone sialoprotein, implying that bone repair is occurring adequately compared to T2D. Studies affirm that resveratrol exerts its osteoprotective effect through osteoblastic differentiation by the SIRT1‐NF‐κB signaling pathway.^43^, ^44^, ^45^, ^46^, ^47^ The activator of this pathway is Rank‐L,^44^ which promotes osteoclast differentiation and activation, regulated by OPG (modulated by the expression of pro‐inflammatory proteins such as IL‐1, IL‐6, IL‐11, and TNF‐α). This fact was confirmed in NGrvt‐NT and T2Drvt‐NT that elevated IBSP expression, as well as in T2Drvt, which was associated with aPDT, where a reduction in TRAP expression was observed. The decreased labeled area by the fluorochromes in T2D‐NT indicates a lower precipitation of mineral matrix in this group, which may be related to lower peri‐implant bone formation in the animals with systemic interference.

In type 2 diabetes, the use of local therapies aided peri‐implant repair, confirming the results of other studies that used aPDT.^41^ However, aPDT without the aid of resveratrol is not able to reverse the damage in cellular events that occur throughout the peri‐implant process, facing the systemic condition evaluated. In this sense, the association of aPDT in T2Drvt animals suggests that the synergism between systemic and local treatment is fundamental to improve bone repair in type 2 diabetic animals. However, in T2D‐aPDT, the use of local therapy promoted an increase in mineral matrix precipitation and a balance in the healing process similar to the normoglycemic groups. Systemic treatment with resveratrol in T2Drvt‐NT promoted an increase in calcein and alizarin precipitation when compared to the other groups. When aPDT was associated with resveratrol in T2Drvt‐aPDT, it can be observed that there was a decrease in calcein precipitation and an increase in alizarin, similar to the NGrvt‐aPDT group.

Fluorochrome labeling is directly related to the precipitation of the mineral matrix in bone tissue.^6^ The NG group showed an increase in the amount of mineral matrix when aPDT was used locally at the time of implant installation, and in addition, the daily MAR was also increased with photonic therapy. This fact explains the increased expression of genes related to osteoclastic activity and the decrease of IBSP in the group since the bone tissue of the group reached mineralization maturity and should initiate bone remodeling.^44^ The NGrvt‐NT group showed a large precipitation of alizarin in bone dynamics, related to the increased expression of genes evaluated in this study, where the use of resveratrol activated pathways related to mineralization as well as to the osteoclastic activity process. When aPDT is used in NGrvt, there is a balance in bone dynamics and the rate of mineral apposition. Thus, the use of resveratrol helped in the process of peri‐implant repair in normoglycemic rats both in the absence of local therapies and in aPDT. As with alveolar bone repair,^6^ mineral matrix precipitation during peri‐implant repair in T2D‐NT animals was impaired, resulting in decreased bone biomechanics. When using aPDT, bone dynamics were restored, a fact that could be explained by the decreased expression of genes related to the bone resorption process and increased expression of genes related to the mineralization process. Systemic use of resveratrol showed a positive effect, this time in the T2Drvt animals, where in NT as well as in aPDT, the animals showed high precipitation of fluorochromes when compared to T2D.

The photosensitization in aPDT transfers electrons and energy to oxygen and the surrounding environment, which can be type 1 or type 2 photosensibilization.^48^ Considering this fact, the electron transfer results in the production of peroxide, hydroxyl radical, and hydrogen peroxide (type 1 photosensibilization), while the energy transfer produces excited singlet oxygen (type 2 photosensibilization).^48^ These reactive oxygen species combine with a variety of molecules that lead to oxidation, degradation, and apoptosis of the cells.^48^ Studies in the literature have shown that the anti‐infectious, anti‐inflammatory, and biostimulator effects^20^, ^21^, ^49^, ^50^ of aPDT post‐exodontic repair. However, few studies have shown the effect of PDT on the osseointegration process.^40^ aPDT has been used in several experimental studies to treat peri‐implantitis and its microorganisms,^51^, ^52^, ^53^, ^54^, ^55^, ^56^ periodontal diseases,^18^, ^19^, ^20^, ^21^, ^22^, ^23^, ^24^, ^51^ and to prevent maxillary osteonecrosis,^21^ proving its efficacy against established dental complications. A previous study of our group demonstrated the beneficial effect of aPDT in reducing *Aggregatibacter actinomycetemcomitans* on tooth sockets of areas with experimental periodontitis in rats.^20^ Studies in animals and in humans have shown beneficial effects of aPDT as a coadjutant in the treatment of peri‐implantitis^42^, ^56^ and periodontal disease in diabetic conditions.^24^, ^51^, ^57^

Variations in this single protocol, leading to several sessions of local therapy or even a longer time of resveratrol administration, possibly would have triggered different results. A single study in dogs^41^ with induced periodontal disease used aPDT preventively before implant installation; however, it followed a different application protocol than the one described in this study. In both studies, equipment of the same brand, same wavelength (660 nm), and same exposure time (60 s) was used; in contrast, the present study used lower energy power and a single point of incidence due to the small size of the rat surgical site. Another important fact is that the choice of photosensitizers was different. While the present study used methylene blue for 60 s before implant installation, the study in dogs used phenothiazine chloride solution for a duration of 5 min. These factors are important to discuss to show the versatility of the use of aPDT, being individualized according to the type of treatment to be performed. The calculated power density was 1.23 W/cm^2^, the absolute power was low (35 mW), and the irradiation time (60 s) was insufficient to induce harmful increases in bone tissue temperature. Thus, the choice of low‐power laser parameters used in this study follows parameters previously studied in bone tissue.^21^ Furthermore, these studies using the same parameters did not reveal tissue damage caused by the choice of time and power used in this model.

Several studies suggest that the use of immediate implant installation protocols associated with decompensated systemic conditions increases the risk of bone loss and implant‐related infections. In the present study, aPDT was used in a single session, prior to implant installation, in an attempt to avoid T2D‐related bone repair complications^6^ by promoting photobiomodulation or microbial reduction effects. It is worth noting that the prophylactic protocol of using aPDT at the time of implant installation has been little explored in the literature^41^ and that in an immediate implant installation model in maxillae, there are external factors that influence peri‐implant bone repair, mimicking the oral conditions that are found in the daily practice of patients. In our study, aPDT was applied locally to the implant site to provide immediate antimicrobial and modulatory effects, while resveratrol was administered systemically to attenuate diabetes‐related oxidative and inflammatory alterations. This distinction highlights that both strategies acted in a complementary rather than competitive manner, contributing to the improved peri‐implant bone healing observed. No previous studies have directly investigated the potential interactions between antimicrobial photodynamic therapy and resveratrol.

Although resveratrol is a natural phytotherapeutic compound with recognized antioxidant properties, its pharmacological interactions are not widely described in the literature. In this context, the present study provides novel insights by evaluating the combined effects of these two approaches in a diabetic model, suggesting that their actions may be complementary rather than antagonistic. Despite promising findings on the effects of resveratrol on bone tissue cells, there are still challenges to translating the osteoprotective effects of resveratrol into clinical applications due to its rapid metabolism and low bioavailability.^58^, ^59^ Our in vivo study showed that, despite benefiting the pathophysiology of type 2 diabetes, resveratrol alone was not able to act on reparative bone biomechanics.

However, it is notable that this is an initial study in our research group that uses these associated therapies in conjunction with the challenge of immediate implant installation in rat maxillae. Another aspect to be considered is that the animals were systemically decompensated, a fact that led to a large implant loss at T2D‐ST, and when local/resveratrol therapies were used, the postoperative success rate was high. The therapies used in this study brought promising results for the prevention of diseases of the oral cavity, increasing the success rate of rehabilitation with titanium implants. So, further studies should be conducted in order to develop new protocols for phototherapies.

## CONCLUSION

Within the limitations of this study, it is possible to conclude that the synergistic effect of resveratrol and aPDT improves the peri‐implant bone biomechanics of type 2 diabetic rats. In addition, the synergistic action of both treatments results in a potential preventive factor for peri‐implant complications commonly found in the treatment of diabetic patients.

## CONFLICT OF INTEREST STATEMENT

None.

## Data Availability

The data that support the findings of this study are available from the corresponding author upon reasonable request.
